# How R&D Staff’s Improvisation Capability Is Formed: A Perspective of Micro-Foundations

**DOI:** 10.3389/fpsyg.2020.551970

**Published:** 2020-09-08

**Authors:** Hui He, Yan Bai, Junguang Gao, Jinqiang Xie

**Affiliations:** ^1^Business School, Beijing Technology and Business University, Beijing, China; ^2^Business School, Beijing Wuzi University, Beijing, China

**Keywords:** improvisation capability, minimal structure, micro-foundations, inverted U-shaped effect, R&D staff

## Abstract

The study examines how R&D staff improvisation capability is formed based on theory of micro-foundations, that is, how R&D individuals, experience and external knowledge gathering, and minimal structure interact and work on their improvisation capability together. The results show that: (1) R&D staff’s experience and external knowledge gathering have linear influences on their improvisation capability, respectively; (2) minimal structure has a curvilinear impact on improvisation capability; (3) minimal structure has a curvilinear moderating effect on the relationship between experience, external knowledge gathering and improvisation capability, respectively. The study suggests that managers keep minimal structure at the moderate level to promote their R&D people’s improvisation capability.

## Introduction

Employee improvisation can be defined as the capability of an individual to deal with complex and unexpected situations in a creative, contextual and professional manner (Weick, 1998; Vera and Crossan, 2005; Hmieleski and Corbett, 2006; Magni et al., 2009; Magni et al., 2010). It seems to have some overlap with individual creativity (Amabile, 1996; Montuori, 2003; Leybourne and Sadler-Smith, 2006; Fisher and Amabile, 2009) and individual innovative behavior (Moorman and Miner, 1998a,b), but represents more dynamic behavior that is necessary in dealing with complex and unexpected conditions. Hence, R&D staff’s improvisation capability is particularly important since their work requires emergent and improvisational approaches to add flexibility and creativity (Niosi, 1999; Cunha and Gomes, 2003).

Antecedents affecting individual improvisation generally involve improvisers’ skills (Vera and Crossan, 2005; Fisher and Amabile, 2009), confidence or self-efficacy (Magni et al., 2010). In addition to individual propensities, the workplace contextual issues have an impact on employee improvisation. For example, organizational structure, culture, team climate and interaction, and managerial practices are captured by a large scale of literatures (Anderson and West, 1998; Cunha et al., 1999; Vera and Crossan, 2004, 2005; Gibbons and Connor, 2005; Lorenz and Lundvall, 2010; Hodgkinson et al., 2016). There still exist some external environmental factors that influence improvisation, e.g., competitive turbulence, market information flows and resources availability (Kyriakopoulos, 2011; Hodgkinson et al., 2016; Yeboah et al., 2016).

Unlike prior scholarly research examining the influencing factors of improvisation capability, our study takes a perspective of micro-foundations of organizational capabilities. Theory of micro-foundations is used to explain how routines and capabilities are formed at a micro level, contributing to the explanation of how organizational competitive advantage can be obtained and sustained (Hoopes and Madsen, 2008). According to micro-foundations, individuals, processes and structure interact and work on organizational capabilities together (Argote and Ingram, 2000; Levinthal and Rerup, 2006; Felin et al., 2012). Organizations can be viewed as an aggregation of individuals, the collection of individual improvisation will therefore make up one of organizational capabilities. The processes refer to the ways that shape routines and capabilities and are usually represented by coordination and technology. Structure reflects the conditions that influence individual or collective processes and interaction. Our study views R&D staff as individuals, experience and external knowledge gathering as process factors, and minimal structure as contextual factor to examine how improvisation capability is formed.

To our knowledge, this study begins to extend the antecedents of improvisation based on theory of micro-foundations. Firstly, we examine the linear relationship between individual experience, external knowledge gathering and improvisation capability. Secondly, we examine the curvilinear relationship between minimal structure and improvisation capability. Finally, we examine the curvilinear moderating effect of minimal structure on the relationship between individual experience, external knowledge gathering and improvisation capability.

The primary theoretical implication lies in an inverted U-shaped effect of minimal structure, which really expands the understanding of its positive side on improvisation linearly (Kamoche and Cunha, 2001; Vera et al., 2014). Also, the study from a perspective of micro-foundations contributes to the explanation of improvisation capability as a source of organizational heterogeneity and competitive advantage (Hoopes and Madsen, 2008). Practically, our study suggests that managers should maintain the important decision-making rights at hand and encourage a stretch of goal setting, so that minimal structure could be kept at the moderate level.

## Theoretical Background and Hypotheses

### Improvisation Capability

Improvisation is usually described as a capacity that can be shown spontaneously in trying to respond to problems or opportunities in a novel way. That implies two things at least: firstly, improvisation is context-related and characterized by spontaneity (Weick, 1993; Hatch, 1998; Vera and Crossan, 2004, 2005), which means an immediate reaction that emphasizes time orientation and an act of being intuitively guided by the situation in a spontaneous way. Secondly, improvisation is purposely and creative, with the deliberate fusion of the design and execution of a novel production (Miner et al., 2001; Helfat et al., 2007).

Recent literature on improvisation capability has moved from arts of jazz, theater and Indian music into organizational field, focusing more on outcomes than antecedent factors of improvisation (Eisenhardt, 1997; Vera and Crossan, 2004; Nemkova et al., 2012). The frequently mentioned outcomes include new product development (Eisenhardt and Tabrizi, 1995; Kyriakopoulos, 2011), innovative performance (Vera and Crossan, 2005; Yeboah et al., 2016), export decision making (Nemkova et al., 2012) and foreign market entry (Bingham, 2009). It is worth to note that improvisation is not inherently good or bad, it does not necessarily produce positive outcomes, which attaches importance to creating a context that supports effective improvisation at workplaces (Vera and Crossan, 2004). Unlike previous study, planning and improvisation are not alternative decision-making orientations but driven by both rational and intuitive reasoning and give rise to responsiveness, namely they may coexist within exporting firms in emerging economy (Nemkova et al., 2012; Nemkova et al., 2015; Hughes et al., 2018b).

Studies on antecedent factors of improvisation capability generally concentrates on the internal characteristics of the organization as well as environmental conditions. Internal characteristics involve individual propensities such as skills, confidence or self-efficacy (Vera and Crossan, 2005; Fisher and Amabile, 2009; Magni et al., 2010), and some organizational traits such as risk-taking, culture, structure, managerial experience and expertise, interactions among members such as real time communication, behavioral integration (Anderson and West, 1998; Cunha et al., 1999; Vera and Crossan, 2004, 2005; Gibbons and Connor, 2005; Lorenz and Lundvall, 2010; Hodgkinson et al., 2016). Environmental conditions refer to uncertainty, competitive turbulence, market information flows, resources availability and they are generally examined as moderating factors (Kyriakopoulos, 2011; Hodgkinson et al., 2016; Yeboah et al., 2016), which is mentioned by Vera et al. (2014) as well.

Improvisation can be viewed as a learning mode (Hughes et al., 2018a). Practicing improvisation capability are more seen in the field of music and dance education, a learner-centered approach and design of activities on processes instead of outcomes are proved to be evident (Biasutti, 2013; Biasutti and Mangiacotti, 2017). Music improvisation program is presented to expand participants’ perceptual skills and motion control, particularly to help in cognitive rehabilitation for older people (Biasutti, 2017, 2015; Biasutti and Mangiacotti, 2017). Improvisation practices can be applied into management domain as well, for example, improvisation training is found to improve new hires’ confidence, ability to adapt in successfully handling unique situations in the airline (Daly et al., 2009). Similarly, improvisation capability can be improved through experimental learning to generate better decision-making in uncertainty for government officials in Brazil (Christopoulos et al., 2016).

### Micro-Foundations of Routines and Capabilities

Routines and capabilities are core concepts in the field of management research, which have played a prominent role in the analysis of organizational and competitive heterogeneity (Hoopes and Madsen, 2008). It is widely accepted that a high level of routine or aggregation of routines make up capability (Winter, 2000, 2003), and capability can strengthen, create or change routines in turn through organizational policies, procedures and structures (Collis, 1994; Helfat et al., 2007).

The issue of why micro-foundations have explicitly entered into agenda of capabilities research deserves considerations. Firstly, the micro-level has a certain explanatory precedence to competitive heterogeneity over macro-level (Abell et al., 2008), since heterogeneity may be located at the individual level and reflect interactions among individuals (Felin and Hesterly, 2006). Secondly, managerial intervention is conducted to maintain competitive advantage. Managers can exert influence on capabilities within organizations by hiring new employees or creating conditions that help to accumulate some certain types of human capital (Abell et al., 2008; Wang and Barney, 2008). Thirdly, research on the micro-level seems to be more stable, fundamental, and prescriptive for poor performance of firms in particular (Coleman, 1990).

Micro-foundations of routines and capabilities can be involved into three core elements: individuals, processes, and structure (Felin et al., 2012). Organization is composed of individuals, and individual characteristics, skills, or cognition are central for organizational outcomes (Simon, 1991; Grant, 1996; Abell et al., 2008). The processes shape routines and capabilities in two fundamental ways: coordination and technology. Whether formal coordination like rules, procedures or informal coordination like experience and norms, they may influence the interdependent actions, critical consequences and capability (Fauchart and von Hippel, 2008; Srikanth and Puranam, 2010). The application of new technologies rebuilds social networks among individuals and influences their learning efficiency (Barley, 1986).

Structure specifies the conditions that enable and constrain individual or collective actions and establish the context for process and interactions. Structure is generally determined by firms, when firms want more flexibility, they make relatively simple rules and policies, leaving room for improvisation; when firms want more control, the rules and policies are becoming more complex to govern activities (Davis et al., 2009). It is noteworthy that even for the same structure, it acts as a double-edged sword. For example, flat structure that encourages great autonomy will bring about more innovation and creativity; however, it will produce obstacles for knowledge sharing within and across sections of organizations (Foss, 2003), which in turn impedes the coordination and capability improvement (Hoopes and Postrel, 1999).

### Formation of R&D Staff’s Improvisation Capability

Since improvisation is viewed as one of organizational capability, it is feasible to examine how improvisation capability is formed based on micro-foundations of capabilities. Considering the three core elements of micro-foundations, we take the R&D personnel as individuals, their experience and external knowledge gathering as process factors in shaping improvisation capability, and minimal structure as contextual factor. As a learning mode, improvisation relies on rapid information collection to carry out an emergent, iterative process, so that better decisions are made under conditions of uncertainty in a novel way (Hmieleski et al., 2013). Information collection may come from internal experience accumulated and external knowledge gathering. That matches the view that improvisation depends on experience and creativity at least (Levinthal and Rerup, 2006).

Contingency theory shows that organizational structure is an important moderator (Nemkova et al., 2012), with formal and centralized structure labeled as mechanistic while informal and decentralized structure as organic (Donaldson, 2001). Improvisation implies that organizational members have freedom in their decision making with little preventing creative and innovative ways of thinking and actions (Brown and Eisenhardt, 1997), that is to say, a more decentralized and flexible structure is preferred. Minimal structure goes beyond decentralization as it allows maximum flexibility within minimum framework (Pasmore, 1998). Besides, minimal structure originates from jazz and provides the context in which actors play in improvising way successfully.

#### Individual Experience and Improvisation Capability

Individual experience refers to the cumulative production history of any one individual, and it is closely linked to task performance (Thorndike, 1898). Traditional researches indicate that the more time individuals take to complete a task, the less errors they make because individuals gain experience with the task. Experience is described to teach individuals how to work more effectively at performing established routines and practices (Reagans et al., 2005). Individual experience plays a crucial role in improvisation capability. Jazz musicians always reorganize their previously performed elements of music into a new melody, similarly, an improviser in an organization could recombine existing elements of routine processes into new types of actions (Hatch, 1998). As individual experience increases, he or she has the opportunity to accumulate more knowledge about the task, which makes it possible to reorganize and integrate existing knowledge from their personal memory to handle with some unexpected events.

Furthermore, individual experience is important even in the team, for individuals have the access to more information about the different roles that they can perform, so that they become more proficient in performing their roles. More importantly, such experience makes individuals know who is good at what and how to make good use of their expertise in teams (Ren and Argote, 2011). Namely, experience teaches team members how to cooperate and produce more efficient division of labor and greater trust and willingness to share knowledge and coordinate specialized roles (Reagans et al., 2005). Also, team members with experience working together may develop further their relationship by enhancing interactions when performing their distinct roles. As the average level of individual experience inside the team increases, team members may improvise more. Hence, we propose H1:

Hypothesis 1 (H1): Individual experience has a significant positive influence on R&D staff improvisation capability.

#### Gathering External Knowledge and Improvisation Capability

According to the knowledge-based approach, the primary role of the firm is identified to integrate the specialist knowledge resident in individuals into goods and services (Grant, 1996). Gathering External Knowledge (GEK) refers to the process of recognizing the value of new external information, assimilating and applying new information into commercial ends. Absorptive capacity is at the core of gathering external knowledge, and it is always related to prior knowledge. Individuals utilize prior related knowledge to assimilate, transfer and then use new knowledge (Cohen and Levinthal, 1990). Therefore, the external knowledge is not only gathered but also assimilated, captured and accumulated. In fact, gathering external knowledge could be described as a creative recombination of external and internal knowledge, even if that external knowledge is only reorganized and assimilated by internal knowledge at one moment and not accumulated for later use (Miner et al., 2001).

The knowledge-based approach offers a theoretical basis for understanding organizational innovations (Grant, 1996). Gathering external knowledge is closely associated with creativity and it may be of great significance for improvisation. Knowledge from external sources has injected new ideas into inherent mind map, igniting creativity spark, and therefore has enhanced individual ability to reconfigure previous experience in new methods (Amabile, 1998; Bresman, 2010). External knowledge is described as novel factors and it can guide individuals to spend more time on projects with greater chance of obtaining a patent (Moorman and Miner, 1998b; Kyriakopoulos, 2011). When individuals are exposed to new knowledge, they can possibly identify current gaps in their internal knowledge and, potential opportunities are therefore recognized to recombine internal and external knowledge for effective improvisation (Vera et al., 2014). Hence Hypothesis 2 is proposed:

Hypothesis 2 (H2): Gathering external knowledge has a significant positive influence on R&D staff improvisation capability.

#### Minimal Structure and Improvisation Capability

Minimal Structure (MS) originates from the jazz improvisation. Unlike other musical forms, jazz contains few strict musical structure such as melody, rhythm, tempo or even composition on performing style and interpretation. Jazz players make use of the structure in improvising ways but they have to comply with a set of musical foundations simultaneously (Hatch, 1997, 1998). These musical foundations show some consensual guidelines and agreements of playing and are conceptualized as minimal structure or semi-structure (Brown and Eisenhardt, 1997). Minimal structure is designed to allow maximum flexibility within a minimum framework of commonality (Pasmore, 1998). In firms, it is also described as the combination of freedom and control (Kamoche and Cunha, 2001). Managers understand the minimal structure in terms of how much control they wish to maintain over their individual members, and how much autonomy they would allow individual members to take the lead at work. In the context of R&D, the level of control usually means how to set one’s own goals and what goal to be prioritized, the level of autonomy decides how to pursue established goals (Vera et al., 2014). Having high autonomy and high goal clarity result in the highest level of minimal structure.

Weick (1989) suggests that great value of minimal structure lies in that it serves to constrain the turbulence of jazz process and lead to creative outcomes of playing. Meyer and Rowan (1977) apply minimal structure firstly into hospitals and schools, they believe that there are clear goals of administration in such organizations and the professionals working inside have some autonomy in performing their tasks. Those doctors and teachers are guided by clear goals and autonomy so that they could experiment, innovate and improvise within wide zones of maneuver (Kamoche et al., 2003). However, high level of minimal structure would probably produce some negative sides of improvisation. For example, over-simplicity or excessive goal clarity may ultimately bring about failure for preventing organizations from adapting to complex and constant changes (Miller, 1993). Similarly, too much autonomy may lead to chaos or confusion which imposes barriers to innovate (Weick, 1989). That implies that managers should maintain the right amount of goal clarity and autonomy, only minimal structure at the moderate level would lead to effective improvisation. The challenge that managers face is how to establish the moderate structure to promote individual improvisation capability. Therefore, we propose Hypothesis 3:

Hypothesis 3 (H3): Minimal structure has an inverted U-shaped relationship with improvisation capability, such that the relationship is positive until an optimal level (threshold) and then becomes negative thereafter.

Prior work has demonstrated that minimal structure could be usually described as a fruitful context favoring the positive links between the knowledge-based processes and improvisation capability (Vera et al., 2014). We have proposed that minimal structure does not necessarily promote improvisation capability, it could have an impact on improvisation capability in a curvilinear way. Likewise, minimal structure could create conditions that influence the links between individual experience, gathering external knowledge and improvisation capability, respectively, in a curvilinear way.

Firstly, minimal structure may create a context for individual experience to influence improvisation capability in a curvilinear way. As mentioned above, individual experience enhances improvisation capability through accumulation and reorganization of knowledge. As minimal structure increases, this knowledge related efforts would be more focused on clear goals, which in turn, may facilitate creativity and innovation for non-redundant knowledge stocks. When individuals have autonomy to handle some inherently ambiguous and complex tasks involved in R&D activities, they would learn more by themselves and thus those experiences may enable to experiment without needing to always seeking management approval (Sundstrom et al., 1990). Even in the context of team, recognizing each one’s expertise could favor role assignment and division of responsibilities, avoiding fragmentation of efforts directed by clear goals. Meanwhile, autonomy allows individuals to risk the comfort of their formal job roles and coordinate in real time in novel ways.

Nevertheless, minimal structure may exert an opposite effect of individual experience on improvisation capability when it goes beyond some optimal level. This is due to two reasons: (1) higher level of goal clarity is not suitable for rapid changes of organizations (Miller, 1993), for it may end in improvisational paralysis by constraining their knowledge integration to few specific issues (Vera et al., 2014); (2) higher level of autonomy may lead to chaotic action in that individual knowledge is accumulated but reorganized disorderly and result in ineffective improvisation (Weick, 1999). We propose Hypothesis 4 accordingly:

Hypothesis 4 (H4): Minimal Structure moderates the relationship between individual experience and improvisation capability in such an inverted U-shaped way that the moderating effect is positive with a low MS until an optimal level (threshold) and then becomes negative thereafter.

Secondly, minimal structure may also moderate the relationship between gathering external knowledge and individual improvisation capability in a curvilinear way. Gathering external knowledge begins with an evaluation of external knowledge, that is the first dimension of absorptive capacity. It is crucial for individuals to have the ability to assess the most valuable external knowledge for assimilation and applying later after they scan new technological developments (Nemanich et al., 2010). The recombination of internal knowledge and the most valuable external knowledge has built the source of improvising creativity. As minimal structure increases, goal clarity creates guidelines for different knowledge sourcing and avoids redundant actions, meanwhile, autonomy provides individuals wide-minded perspectives to gather and recombine external knowledge. Therefore, minimal structure with high level of goal clarity and autonomy channels external knowledge gathering and then enriches the improvisation capability.

Like the negative effect of individual experience on improvisation capability, gathering external knowledge may have the similar impact on improvisation capability if minimal structure could not maintain the right size. Clearer goal could lead to a death of creativity by restricting the scope of knowledge gathering from external sources, and more autonomy could result in turbulence in improvisation for misuse of resources. Thus, we suggest the following Hypothesis 5:

Hypothesis 5 (H5): Minimal Structure moderates the relationship between gathering external knowledge and improvisation capability in such an inverted U-shaped way that the moderating effect is positive with a low MS until an optimal level (threshold) and then becomes negative thereafter.

**FIGURE 1:**
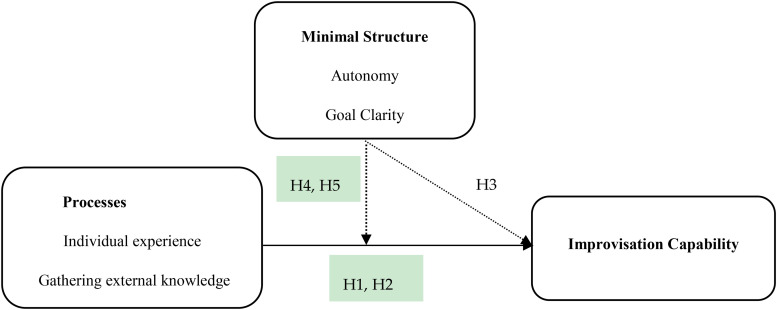
Research Model (full line means linear relation while imaginary line means non-linear relations).

Figure 1 shows our research model.

## Materials and Methods

### Sample and Data Collection

A questionnaire survey was conducted to collect data from July to November, 2017, targeting at R&D members of 5 firms in software development and information service in Beijing and Tianjin. We sent these questionnaires through firms’ human resource departments. In order to avoid common method variance, we conducted 2 formal surveys for each firm with an interval of 1 month. The first survey was distributed to 254 R&D staff to measure demographics, individual experience and gathering external knowledge. A month later, the second survey was distributed to the same groups of respondents to measure minimal structure and improvisation capability. 210 matched data were found available for final analysis.

Of the respondents, 67.9% were male, 32.1% were female; 27.5% were from 20 to 25 years of age, 36.3% were from 26 to 30 years of age; 20.7% were from 31to 35 years of age; 12.4%were from 36 to 40 years of age; 3.1%were aged over 40. According to the education background, the distribution of junior college and below accounts for 12.4%, bachelor’s for 59.6%, master and above accounts for 28%; 39.9% were those whose working years here is from 1 to 3 years; 19.7% from 3 to 5 years; 11.9% from 5 to 7 years; 28.5.% were from 7 years and beyond.

This study was approved by the local ethics committee, and all participants provided written informed consent after complete the study. Also, guarantees of confidentiality and anonymity were provided to respondents to reduce respondent anxiety or answers based on social desirability.

### Study Measures

#### Variable Measurement

All measurement scales are drawn from previous studies and align with the definitions of the constructs examined. To ensure the congruence of English versions of the scales, Chinese versions were firstly translated by the first author, and then the Chinese versions were back translated by a separate party. And no major discrepancies were detected in the back translation. Finally, wording for certain measures was adjusted in minor ways to enhance the readability in China. Items were rated on a scale that ranged from “strongly disagree” (1) to “strongly agree” (7) and were averaged to form an index for each employee.

Improvisation Capability (IC) is a higher-order factor created from measures of creativity (CE) and spontaneity (SO). It was measured with 6 items developed by Vera and Crossan (2005), 3 items for spontaneity and 3 for creativity, the typical item is “We could deal with unanticipated events on the spot.”

Likely, Minimum Structure (MS) was measured by a combination of task autonomy and goal clarity (Kamoche et al., 2003). Autonomy (AT) refers to the capability to act autonomously without the approval of management, for example, “Without seeking management approval, we could make our own decisions about the innovation design.” Goal Clarity (GC) refers to the extent to which the R&D staff objectives are clearly defined at a certain point, such as “From the beginning of any invention project, we were very clear about its goals.”

Individual experience (IE) involves the technical knowhow accumulated by any one individual on projects, and his or her awareness of each other’s strengths and weaknesses, which was measured with six items (Argote and Ingram, 2000; Tesluk and Jacobs, 2010), for example, “we could make good use of technical knowledge accumulated on various projects,” “We know what other members of the team are good at and what they are not.”

Gathering external knowledge (GEK) was measured with a two-item scale developed by Vera et al. (2014), for example, “we were very capable at gathering information about new technological developments originating outside our company.”

All measures were subjected to confirmatory factor analysis (CFA). The properties of these measurement items as derived through CFA are presented in Table 1 and construct robustness and descriptive statistics are presented in Table 2.

**TABLE 1:** Measurement item properties.

**Construct**	**Measurement item**	**Factor loading**	***t*-value**
SO	We could deal with unanticipated events on the spot.	0.817	11.829
	We could think on their feet when carrying out actions.	0.798	12.690
	We could respond in the moment to unexpected problems.	0.86	12.289
CE	We could identify opportunities for new work processes.	0.823	10.842
	We could take risks in terms of producing new ideas in doing its job.	0.743	10.612
	We could demonstrate originality in our work.	0.733	9.638
AT	Without seeking management approval, we could experiment freely while designing the innovation.	0.833	12.503
	Without seeking management approval, we could make our own decisions about the innovation design.	0.823	12.816
	Without seeking management approval, we could try out our ideas for the innovation design.	0.861	12.297
GC	From the beginning of this invention project, we were very clear about its objectives.	0.79	11.842
	From the beginning of this invention project, we were given clear guidance on what our top priority objectives should be.	0.722	11.703
	From the beginning of this invention project, we had a firm understanding about the objectives we were expected to achieve.	0.847	10.567
IE	We were very competent at pooling some valuable new technical knowledge.	0.764	10.484
	We could make good use of technical knowledge accumulated on various projects.	0.864	11.933
	We were skillful at recombining previous technical knowledge for new projects.	0.759	10.403
	We know what other members are good at and what they are not.	0.756	10.366
	I have worked with other team members before.	0.633	5.706
	We all know about ways to work together better.	0.763	12.071
GEK	With respect to new technological developments originating outside our company, we were very capable at gathering news about them.	0.825	6.674
	With respect to new technological developments originating outside our company, we were very skillful at finding out about them.	0.788	7.674

*SO, spontaneity; CE, creativity; AT, autonomy; GC, goal clarity; IE, individual experience; GEK, gathering external knowledge.*

**TABLE 2:** Reliability and construct robustness.

	**α**	**CR**	**AVE**	**SO**	**CE**	**AT**	**GC**	**IE**	**GEK**
**SO**	0.866	0.865	0.681	0.825					
**CE**	0.810	0.811	0.589	0.677***	**0.767**				
**AT**	0.877	0.877	0.704	0.485***	0.571***	**0.839**			
**GC**	0.844	0.830	0.621	0.659***	0.711***	0.452***	**0.788**		
**IE**	0.865	0.890	0.577	0.691***	0.758***	0.535***	0.703***	**0.760**	
**GEK**	0.788	0.788	0.651	0.337***	0.451***	0.382***	0.378***	0.438***	**0.801**

(1) SO, spontaneity; CE, creativity; AT, autonomy; GC, goal clarity; IE, individual experience; GEK, gathering external knowledge; (2) α, Cronbach alpha; CR, construct reliability; AVE, average variance extracted; Bolded numbers on the diagonals represent the square root of AVE; (3) Significance level: ***p < 0.001.

#### Reliability and Validity Tests

All construct reliability and average variance extracted values are above accepted thresholds. α values of all variables were ranging from 0.788 to 0.877, indicating that all the scales had acceptable reliability. Convergent validity is demonstrated as the path coefficients from each measurement item to their respective latent variable are statistically significant as all items load significantly. As shown in Table 2, the square root of average variance extracted for each construct exceed the correlation values between that construct and all other constructs, and so discriminant validity is confirmed. Taken together, these provide strong evidence for construct validity.

#### Measurement Model

Furthermore, we present the outcome of a robust maximum likelihood analysis on the full measurement model provides a set of model fit indexes and a robust chi-square statistic (see Table 3). The results clearly showed that a three-factor measurement model (Individual experience + Gathering external knowledge, Goal clarity + Autonomy, Improvisation Capability) has a very good fit with the data, with χ2 = 265.236, RMSEA = 0.057, CFI = 0.962, GFI = 0.89, AGFI = 0.85, NFI = 0.903, TLI = 0.953. Therefore, three-factor model is used in the assessment of the construct.

**TABLE 3:** Assessment of the measurement model.

**Factor structure**	**χ2**	**df**	**χ^2^/df**	**RMSEA**	**CFI**	**GFI**	**AGFI**	**NFI**	**TLI**
One-factor model	418.262	149	2.807	0.101	0.873	0.813	0.762	0.817	0.854
Two-factor model^a^	380.829	147	2.591	0.095	0.890	0.821	0.769	0.834	0.872
Two-factor model^b^	375.419	148	2.537	0.093	0.893	0.821	0.771	0.836	0.876
Three-factor model	265.236	143	1.767	0.057	0.962	0.89	0.85	0.903	0.953

*One-factor model (individual experience + gathering external knowledge + *goal clarity* + *autonomy* + *improvisation capability); two-factor model a (individual experience* + *gathering external knowledge* + *goal clarity* + *autonomy, improvisation capability); two-factor model b (individual experience* + *gathering external knowledge, goal clarity* + *autonomy* + *improvisation capability); three-factor model (individual experience* + *gathering external knowledge, goal clarity* + *autonomy, improvisation capability).**

## Results

### Descriptive Statistics and Correlations

The descriptive statistics and correlation coefficients among all the variables the study used are presented in Table 4. Individual experience and gathering external knowledge were found to be significantly related to improvisation capability (*r* = 0.690, *p* < 0.01; *r* = 0.428, *p* < 0.01). Goal clarity and autonomy were found to be significantly related to improvisation capability (*r* = 0.647, *p* < 0.01; *r* = 0.574, *p* < 0.01). These results support the hypothesized relations regarding individual experience, gathering external knowledge and improvisation capability.

**TABLE 4:** Correlations analysis and means, standard deviations.

**Constructs**	***M***	***SD***	**IE**	**GEK**	**IC**	**AT**	**GC**
IE	4.9263	1.13293	1				
GEK	4.7458	1.18792	0.438**	1			
IC	4.9106	1.06679	0.690**	0.428**	1		
AT	4.0857	1.36986	0.535**	0.382**	0.574**	1	
GC	5.0559	1.27175	0.603**	0.378**	0.647**	0.452**	1

(1) IE, individual experience; GEK, gathering external knowledge; IC, improvisation capability; AT, autonomy; GC, goal clarity. (2) Significance level: **p < 0.01.

### Regression Analysis

Multiple regression analysis is used to test the hypotheses in SPSS Statistics 20. In undertaking the regression analysis, a 3-step sequence is followed. The control variables and the direct effects of individual experience and gathering external knowledge are firstly tested on R&D staff’s improvisation capability, a linear regression is used therefore. Then we examine the curvilinear relationship between minimal structure and improvisation capability, finally the interaction item of individual experience, gathering external knowledge and square of minimal structure are introduced to test an inverted U-shaped moderating effect. All of the results are shown in Tables 5, 6.

**TABLE 5:** Regression analysis for individual experience on improvisation capability.

**Variable model**	**Improvisation capability (IC)**										
	**M1**	**M2**	**M3**	**M4**	**M5**	**M6**	**M7**	**M8**	**M9**	**M10**	**M11**
Intercept	4.885***	0.886**	0.478	0.347	2.967*	0.527	0.803	1.388	0.653*	0.774	2.693*
Gender	−0.098	−0.093	−0.059	−0.060	−0.210	−0.042	−0.041	−0.13	−0.076	−0.076	−0.13
Age	0.047	0.063*	−0.018	−0.018	0.038	0.025	0.024	0.049	0.01	0.009	0.021
Education	0.012	0.136	0.129*	0.130*	0.138*	0.160+	0.157	0.166*	0.097	0.095	0.023
Work years	0.084	−0.007	0.048	0.047	0.044	0.043	0.044	−0.037	0.026	0.027	0.018
IE		0.752***	0.463***	0.492***	0.831*	0.644***	0.589***	0.667**	0.508***	0.479***	0.447**
MS			0.396***	0.430***	0.575**						
AT						0.150**	0.066	0.304			
GC									0.304***	0.277*	0.776
IE*MS				0.007	0.025**						
IE*MS^2^					−0.041**						
IE*AT							0.017	0.031*			
IE*AT^2^								−0.035*			
IE*GC										0.006	0.084+
IE*GC^2^											−0.03*
R^2^	0.019	0.641	0.426	0.426	0.450	0.511	0.511	0.649	0.545	0.545	0.704
AdjustedR^2^	0.009	0.628	0.402	0.399	0.421	0.491	0.488	0.628	0.531	0.528	0.692
R^2^Variation	0.019	0.623	0.407	0	0.024	0.492	0.001	0.138	0.704	0	0.159
F	0.683	51.081***	18.105***	15.750***	15.366***	25.519***	22.245***	31.083***	40.338***	50.526***	58.055***

(1) IE, individual experience; GEK, gathering external knowledge; IC, improvisation capability; AT, autonomy; GC, goal clarity; MS, minimal structure; (2) Significance level: ***p < 0.001, **p < 0.01, *p < 0.05, ^+^p < 0.1.

**TABLE 6:** Regression analysis for gathering external knowledge on improvisation capability.

**Variable**	**Improvisation capability (IC)**										
**Model**	**M1**	**M2**	**M3**	**M4**	**M5**	**M6**	**M7**	**M8**	**M9**	**M10**	**M11**
Interception	4.885***	3.073***	1.154**	0.233	0.428	2.118***	1.103	3.213*	1.319***	0.673	4.047**
Gender	−0.098	−0.085	−0.067	−0.089	−0.058	−0.079	−0.091	−0.066	−0.059	−0.679	−0.091
Age	0.047	0.169	0.041	0.050	0.062	0.046	0.021	0.027	0.003	0.001	0.015
Education	0.012	−0.05	0.050	0.048	0.017	0.035	0.025	0.029	−0.009	−0.004	0.007
Work years	0.084	−0.008	0.067	0.069	0.057	0.036	0.042	0.018	0.056	0.056	0.034
GEK		0.396***	0.226*	0.344*	0.218*	0.257***	0.486**	0.447**	0.151**	0.302*	0.554*
MS			0.685***	0.834***	0.638**						
AT						0.378***	0.707***	0.875			
GC									0.570***	0.719***	0.625
GEK*MS				0.054	0.005*						
GEK*MS^2^					−0.014*						
GEK*AT							0.067	0.052*			
GEK*AT^2^								−0.046*			
GEK*GC										0.033	0.036**
GEK*GC^2^											−0.055**
*R*^2^	0.019	0.204	0.509	0.515	0.524	0.397	0.409	0.423	0.588	0.591	0.614
Adjusted *R*^2^	0.009	0.176	0.489	0.492	0.499	0.372	0.381	0.389	0.571	0.571	0.591
*R*^2^ variation	0.019	0.186	0.305	0.006	0.009	0.378	0.012	0.014	0.57	0.003	0.023
***F***	0.683	7.342***	25.287***	22.531***	20.667***	16.088***	14.691***	12.323***	34.795***	30.665***	26.714***

(1) IE, individual experience; GEK, gathering external knowledge; IC, improvisation capability; AT, autonomy; GC, goal clarity; MS, minimal structure; (2) Significance level: ***p < 0.001, **p < 0.01, *p < 0.05.

As shown in Table 5, we examine the effect of individual experience on improvisation capability firstly. Individual experience has significant positive associations with improvisation capability, see model 2 (*b* = 0.752, *p* < 0.001), H1 is supported. Similarly in Table 6, gathering external knowledge has significant positive associations with improvisation capability, see model 2 (*b* = 0.396, *p* < 0.001), H2 is also supported.

In order to test hypothesis 3, we use a formula to examine the inverted U-shaped relationship between the minimal structure and improvisation capability (Haans, 2016):

Y⁢i⁢t=β⁢1⁢X⁢i⁢t+β⁢2⁢X⁢2⁢i⁢t+β⁢3⁢Z⁢i⁢t+ε⁢i⁢t

In this formula, Yit represents the explained variable, that is improvisation capability. Xit represents the explanatory variable, that is minimal structure. X2it means the quadratic term of the explanatory variable, β1 means the regression coefficient of explanatory variable, and β2 represents the regression coefficient of the square of explanatory variable. Zit is a vector of control variables, β3 is a vector of regression coefficients, and εit is an error term. If there is an inverted U-shaped relationship between Xit and Yit, the following three criteria must be met simultaneously (Haans, 2016): (1) β2 is significantly negative; (2) If XL represents the minimum of Xit, XH represents the maximum of Yit, then the slope at XL (β1 + 2β2XL) should be positive and the slope at XH (β1 + 2β2XH) be negative (3) The inflection point of the formula (−β1/2β2) should be within the value range of Xit.

We follow the three criteria above to test H3, using hierarchical regression analysis (see Table 7). Model 1 includes control variables. Model 2 adds MS and Model 3 adds the squared term of MS subsequently. As shown in Model 3, minimal structure has significant positive associations with improvisation capability (IC) (*b* = 0.539, *p* < 0.001), and the squared term of MS is negative and significant (*b* = −0.045, *p* < 0.05), which has met the first criteria. Furthermore, the slope of the curve is positive at the left end (β1 + 2β2XL = 0.4193) and the slope of the curve is negative at the right end (β1 + 2β2XH = −0.0757). The inflection point (−β1/2β2 = 5.9889) is at the value range of the minimal structure indeed. It is seen that the three criteria have been satisfied and minimal structure therefore has an inverted curvilinear relationship with improvisation capability, supporting fully H3. We proceed to examine the two dimensions of minimal structure. Autonomy has significant positive associations with improvisation capability (*b* = 0.467, *p* < 0.001), and its squared term is negative significantly in Model 5 (*b* = −0.062, *p* < 0.1). The slope of the curve is positive at the left end (β1 + 2β2XL = 0.343) and negative at the right end (β1 + 2β2XH = −0.401). The inflection point (−β1/2β2 = 3.166) is within the value range of the autonomy. Likewise, goal clarity has significant positive associations with improvisation capability and its squared term negative (*b* = 0.494, *p* < 0.001; *b* = −0.042, *p* < 0.1). The slope is positive at the left end (β1 + 2β2XL = 0.382) and it is negative at the right end (β1 + 2β2XH = −0.494). The inflection point (−β1/2β2 = 5.881) falls within the value range of goal clarity.

**TABLE 7:** The analysis of the inverted U-shaped relationship.

**Variable**	**Improvisation capability (IC)**						
**Model**	**M1**	**M2**	**M3**	**M4**	**M5**	**M6**	**M7**
Intercept	4.885***	1.215***	0.410	1.693***	0.509	1.536***	0.693***
Gender	−0.098	−0.095	−0.102	−0.172	−0.091	−0.133	−0.132
Age	0.047	0.071	0.077	0.011	0.014	0.06	0.065
Education	0.012	0.041	0.040	0.071	0.098	−0.009	−0.003
Working years	0.084	0.072	0.067	0.144	0.135	0.108	0.101
MS		0.743***	0.539***				
MS^2^			−0.045*				
AT				0.512**	0.467***		
AT^2^					−0.062^+^		
GC						0.6***	0.494***
GC^2^							−0.042^+^
*R*^2^	0.019	0.610	0.616	0.361	0.373	0.402	0.408
Adjusted *R*^2^	−0.009	0.603	0.607	0.339	0.347	0.391	0.395
*F*	0.683	81.446***	71.262***	16.014***	14.382***	34.984***	30.630***

(1) IE, individual experience; GEK, gathering external knowledge; IC, improvisation capability; AT, autonomy; GC, goal clarity; MS, minimal structure; (2) Significance level: ***p < 0.001, **p < 0.01, *p < 0.05, ^+^p < 0.1.

Then we try to test “inverted U-shaped” moderating effect of minimal structure: firstly, we construct the direct effect of individual experience and gathering external knowledge on improvisation capability; next, we verify the linear moderating role of minimal structure; finally, the interaction item of individual experience (or gathering external knowledge) and square of minimal structure are introduced to verify our hypotheses.

From model 4 in Table 7, we can see that the relationship between individual experience and improvisation capability is not moderated by minimal structure (*b* = 0.007, *p* > 0.1). More specifically, neither autonomy nor goal clarity plays a moderating role between individual experience and improvisation capability (*b* = 0.017, *p* > 0.1; *b* = 0.006, *p* > 0.1). We conclude that minimal structure has no obvious linear moderating effect, it may have a curvilinear effect. Model 5 indicates that the interaction item of the individual experience and the square of minimal structure is negatively significant (*b* = −0.041, *p* < 0.01), which means that minimal structure has a significant “inverted U-shaped” moderating effect on the relationship between individual experience and improvisation capability, H4 is supported. More specifically, we test the “inverted U-shaped” moderating effect of autonomy and goal clarity separately. Model 8 indicates that the interaction item of the individual experience and the square of autonomy is negatively significant (*b* = −0.035, *p* < 0.05), model 11 indicates that the interaction item of the individual experience and the square of goal clarity is negatively significant as well (*b* = −0.03, *p* < 0.05), implying that both autonomy and goal clarity exert an “inverted U-Shaped” moderating effect on the relationship between individual experience and improvisation capability.

Similarly, minimal structure does not moderate the relationship between gathering external knowledge and improvisation capability (*b* = 0.054, *p* > 0.1). More specifically, neither autonomy nor goal clarity plays a moderating role between gathering external knowledge and improvisation capability (*b* = 0.067, *p* > 0.1; *b* = 0.033, *p* > 0.1) which indicate that the minimal structure has insignificant linear moderating effect. And then Model 5 indicates that the interaction item of the gathering external knowledge and the square of minimal structure is negatively significant (*b* = −0.014, *p* < 0.05), which indicates that minimal structure has an “inverted U-shaped” moderating effect on the relationship between gathering external knowledge and improvisation capability, H5 is supported. More specifically, the interaction item of the gathering external knowledge and the square of autonomy is negatively significant in Model 8 (*b* = −0.046, *p* < 0.05), and the same thing happens to goal clarity in Model 11 (*b* = −0.055, *p* < 0.01), meaning that both autonomy and goal clarity have significant “inverted U-shaped” moderating effect on the relationship between gathering external knowledge and improvisation capability.

## Discussion

Based on micro-foundations and use of a large sample of empirical research, we examine the formation of R&D staff improvisation capability and several results are as followed:

Firstly, we find that both individual experience and external knowledge gathering can promote R&D members’ improvisation capability. These results are completely consistent with previous studies and support the view that improvisation arises from experience and creativity at least (Levinthal and Rerup, 2006).

Secondly, our study shows that minimal structure has a significant curvilinear impact on R&D staff improvisation capability. Most of prior literature recognizes the active role that minimal structure plays in improvisation, our study reveals the double-edged sword effect of minimal structure which is mentioned but not verified before. By use of a large amount of effective samples, we suggest that minimal structure has an inverted U-shaped relationship with improvisation capability, such that R&D personnel provided with lower and higher levels of autonomy and goal clarity will have less improvisation capability than those provided with moderate levels of task autonomy and goal clarity. This result reflects a critical point existing between the positive and negative side of autonomy and goal clarity on improvisation capability.

Lastly, an inverted U-shaped moderating effect of minimal structure is therefore concluded on the relationship between individual experience, external knowledge gathering and improvisation capability, respectively. This is a further verification of inverted U-shaped effect of minimal structure. Specifically, when R&D members are presented with a lower level of task autonomy and goal clarity, their individual experiences and external information gathering could positively influence their improvisation capabilities. But there exists a threshold for this positive relationship and thereafter such relationship becomes negative with higher level of autonomy and goal clarity. This finding shows that the impact of minimal structure is more complex and there actually exists an optimal level where R&D members improvise most.

## Theoretical and Practical Implications

Based on the theory of micro-foundations, we examine how individuals, processes and structure interact and work on R&D members’ improvisation capability together. Prior study on influencing factors of improvisation generally concentrates on the internal characteristics and external conditions of organizations, our study contributes more to the explanation of improvisation capability as one of organizational heterogeneity and competitive advantage (Hoopes and Madsen, 2008). That provides theoretical grounds and empirical evidences that some managerial intervention at micro-level can be introduced into organizations so that improvisation capability can be enhanced, and furthermore, organizational competitive advantage can be gained and sustained.

In addition, we verify the curvilinear effect of minimal structure, which really expands the existing research that always emphasizes its positive side on improvisation linearly (Kamoche and Cunha, 2001; Vera et al., 2014). Our findings reveal that there would be an optimal level of minimal structure where R&D staff would improvise most, and where experience and gathering external knowledge would have the strongest effect on R&D staff’s improvisation capability. Our study provides important evidence that minimal structure seems to be complex and full of contingency. By examining minimal structure as moderator, this study expands to the comprehension of the boundary conditions of the relationship between experience, gathering external knowledge and improvisation capability, respectively.

The primary managerial contribution lies in that our study advocates experience accumulation and external knowledge collection as two potential ways to promote R&D members’ improvisation capability. Managers should carefully motivate R&D staff to participate in various projects across sections to obtain more experience and gather more external knowledge, so that R&D people will improvise more to deal with some uncertainty. Considering the inverted U-shaped effect of minimal structure, we suggest managers who seek to increase their members’ improvisation should keep minimal structure at the moderate level. Managers can establish clear goals which their members’ efforts are focused on and grant their self control over their tasks. But the obvious danger is that goal clarity and self control can easily increase to a level that is detrimental rather than beneficial. Therefore, managers should keep the important decision-making rights at hand and also encourage a stretch of goal setting, which contribute to preventing much higher level of minimal structure. For example, managers should create an open-minded information exchange channels through which clear goals but with some flexibility can be achieved. In addition, managers can intentionally maintain frequent contacts with researchers so that their progress can be tracked.

## Limitations and Future Research Directions

It has a great value to look at how improvisation capability is developed since it is viewed as a learning mode. Nevertheless, our study design did not allow us to capture the evolution of this capability. Longitudinal or qualitative work would enable the examination of the dynamic aspects of our study in the future. Our data collection from 23 teams of 5 firms, it is almost impossible to make multilevel analysis even though minimal structure acts as a contextual factor in a higher level. Future study would further examine the impact of contextual factor from multilevel aspect. Also, we have to mention the gender imbalance of sampling since our male samples are much greater more than female samples. It reflects the fact that men represent a majority in R&D field in China, yet that may have some impacts on their improvisation capability, so gender difference would be further considered in future study on improvisation capability.

Our study measures minimal structure by a combination of autonomy and goal clarity, trying to reflect “maximum freedom within minimum boundaries” was achieved at higher levels of both. However, minimal structure was inherently paradoxical and dialectic (Weick, 1998), for the two measures of freedom and control were negatively correlated and envisioned as endpoints of the same continuum (Vera et al., 2014). New studies would identify the optimal level of freedom versus control by calculating the inflection point in the inverted U-shaped curvilinear model.

Additionally, our study incorporated experience and creativity toward improvisation capability and found their positive functions, respectively. From my point of view, the coexistence of “old” knowledge from individual experience and “new” knowledge from external sources complies with the balance between exploration and exploitation in improvising. This is explained that R&D people can explore and develop new solutions to unexpected problems or situations by exploiting and recombining current methods and processes. Further studies could examine how experience and creativity interact and work together on improvisation.

## Data Availability Statement

The raw data supporting the conclusions of this article will be made available by the authors, without undue reservation, to any qualified researcher.

## Ethics Statement

Ethical review and approval was not required for the study on human participants in accordance with the local legislation and institutional requirements. Written informed consent from the (patients/participants or patients/participants legal guardian/next of kin) was not required to participate in this study in accordance with the national legislation and the institutional requirements.

## Author Contributions

HH: conceptualization, methodology, validation, original draft preparation, resources, supervision, and funding acquisition. YB: methodology, software, review, editing, and visualization. JG: methodology, data curation, review, and editing. JX: supervision. All authors contributed to the article and approved the submitted version.

## Conflict of Interest

The authors declare that the research was conducted in the absence of any commercial or financial relationships that could be construed as a potential conflict of interest.
